# Supplementary material for: Analyzing scRNA-seq data by CCP-assisted UMAP and tSNE
Source: PLoS One. 2024 Dec 13;19(12):e0311791. doi: 10.1371/journal.pone.0311791 (PMC11642954; doi:10.1371/journal.pone.0311791)
Supplement: S1 File — (PDF) [file pone.0311791.s001.pdf]

Supporting Materials for Analyzing scRNA-seq data by CCP-assisted UMAP and t-SNE

Yuta Hozumi[1] and Guo-Wei Wei[1,2,3*]
[1] Department of Mathematics,
Michigan State University, MI 48824, USA.
[2] Department of Electrical and Computer Engineering,
Michigan State University, MI 48824, USA.
[3] Department of Biochemistry and Molecular Biology,
Michigan State University, MI 48824, USA.

September 30, 2024

Contents

S1 Methods **2**
S1.1 Gene clustering . . . 2

S2 Results **2**
S2.1 Data statistics . . . 2
S2.2 Comparison of CCP, PCA and NMF assisted visualization . . . 3
S2.3 Accuracy . . . 7

S3 Discussion **10**
S3.1 Large Data . . . 10
S3.2 Low variance gene . . . 11

keywords: scRNA-seq, dimensionality reduction, machine learning, visualization

*Corresponding author. Email: weig@msu.edu

S1 Methods

S1.1 Gene clustering

CCP implements k-means clustering to partition the genes. Let $\mathcal{Z} = \{\mathbf{z}^i\}_{i=1}^I$ be the gene vectors. In k-means clustering, the goal is to partition $\mathcal{Z}$ into $\mathcal{Z}^1, ..., \mathcal{Z}^N$, where $N << I$.

K-means clustering begins by randomly selection N centroids, or the center of the clusters. The centroids are denoted as $\mu_1, ..., \mu_N$, and μ_n is the center of cluster $\mathcal{Z}^n$. Using the randomly selected centroids, each gene is assigned to the nearest centroid, which creates the initial clusters. Then, the centroids and the cluster assignment is updated by minimizing the within cluster sum of squares (WCSS).

$$\sum_{n=1}^{N} \sum_{\mathbf{z}^i \in \mathcal{Z}^n} \|\mathbf{z}^i - \mu_n\|^2 \tag{1}$$

This gives the updating scheme for the centroids

$$\mu_n = \frac{1}{|\mathcal{Z}^n|} \sum_{\mathbf{z}^i \in \mathcal{Z}^n} \mathbf{z}^i, \tag{2}$$

where $|\mathcal{Z}^n|$ is the number of genes in partition $\mathcal{Z}^n$. This updating scheme is repeated until convergence or until the maximum number of iterations is reached.

Let $S = \{1, ..., I\}$ be the enumeration of the genes. We can partition S into $S^1, ..., S^N$ using the partitioning results from before by stetting $S^n = \{i|\mathbf{z}^i \in \mathcal{Z}^n\}$. Using this partition, we can denote $\mathbf{z}_m^{S^n}$ to be the genes S^n of the m-th cell, which will be utilized in the gene-projection step.

S2 Results

S2.1 Data statistics

Table S1 show basic statistical analysis of the dataset, namely the sparsity (number of zero expression), max, mean and median expression, and the median sum of the cell expression. For the mean and the median expression, we considered all nonzero expression values.

Table S1: Accession ID, source organism, and the counts for samples, genes, and cell types for fourteen individual datasets

Dataset	Organism	Size (cells x genes)	Sparsity	Max	Mean	Median	Median Row Sum
GSE75748cell	Human	1018 x 19097	49.64	605598.59	470.98	75.00	4404343.22
GSE75748time	Human	758 x 19189	54.69	165308.35	153.61	27.00	1306562.14
GSE82187	Mouse	705 x 18840	77.72	5.60	1.70	1.73	7086.69
GSE94820	Human	1140 x 26593	80.72	600749.50	195.06	17.38	999999.98
GSE67835	Human	420 x 22084	81.40	58272.00	136.71	27.00	505256.00
GSE84133human1	Human	1937 x 20125	90.44	4318.00	3.02	1.00	5346.00
GSE84133human2	Human	1724 x 20125	90.59	3476.00	2.70	1.00	4889.50
GSE84133human3	Human	3605 x 20125	91.30	3071.00	3.35	1.00	4742.00
GSE84133human4	Human	1303 x 20125	89.05	4234.00	3.05	1.00	6017.00
GSE84133mouse1	Mouse	822 x 14878	90.48	3477.00	2.64	1.00	2936.50
GSE84133mouse2	Mouse	1064 x 14878	87.81	3656.00	3.28	1.00	5631.00
GSE84133 human	Human	8569 x 20125	90.62	4318.00	3.09	1.00	5075.00
Muraro	Human	2122 x 19046	73.02	4501.60	3.52	1.60	18076.53
Romanov	Mouse	2881 x 21143	85.92	2571.46	2.48	1.26	7373.00
Qx Bladder	Mouse	2500 x 23341	86.94	4640.55	3.64	1.27	11102.50
Qx Limb Muscle	Mouse	3909 x 23341	93.57	1124.88	2.74	1.17	4110.00
Qx Spleen	Mouse	9552 x 23341	94.34	1665.24	2.46	1.07	3244.50
Qs Diaphragm	Mouse	870 x 23341	91.35	481734.98	247.92	70.45	500405.50
Qs Limb Muscle	Mouse	1090 x 23341	89.47	682914.40	294.23	78.39	723268.50
Qs Lung	Mouse	1676 x 23341	89.08	300415.15	245.66	67.56	626066.50
Qs Trachea	Mouse	1350 x 23341	85.48	730410.52	220.08	61.81	745632.50
SCP1749	Primate	10006 x 24820	94.28	1084.24	1.29	0.71	1289

S2.2 Comparison of CCP, PCA and NMF assisted visualization

In this section, we show the effectiveness of CCP as an initialization of UMAP and tSNE by comparing it to PCA and NMF, 2 of the most utilized algorithm for dimensionality reduction for scRNA-seq data. We perform the same preprocessing procedure as described in Section 3.1 of the main text.

Figure S1 show the comparison of CCP-assisted, PCA-assisted and NMF-assisted visualization on GSE75748 cell, GSE75748 time, GSE67835 and GSE82187 data. The columns from left to right correspond to CCP-assisted UMAP, CCP-assisted tSNE, PCA-assisted UMAP, PCA-assisted tSNE, NMF-assisted UMAP and NMF-assisted tSNE.

NMF-assisted visualization do not provide a meaningful clustering result for all data. We can observe the following from the visualization:

- In GSE75748 cell, CCP-assisted and PCA-assisted visualization show similar result.
- In GSE75748 time data, CCP-assisted and PCA-assisted visualization show similar result. Most notably, both technique show a supercluster of 72hr and 96hr, which is consistent with Chu's findings [1]. There is a clearer distinction between the 72hr-96hr supercluster with 36hr cluster for PCA-assisted visualization. However, CCP-assisted visualization show a clear distinction of 00hr cells from other cells, indicating that there is a significant difference between the undifferentiate cells from the other cells.
- In GSE67835 data, CCP-assisted UMAP and tSNE have the best visualization. PCA-assisted UMAP do no show clear clustering, and PCA-assisted tSNE visualization is dominated by an outlier astrocytes.
- Both CCP-assisted UMAP and tSNE show the best visualization for GSE82187 data. All the cell types

show a distinct cluster, whereas PCA-assisted visualization do not.

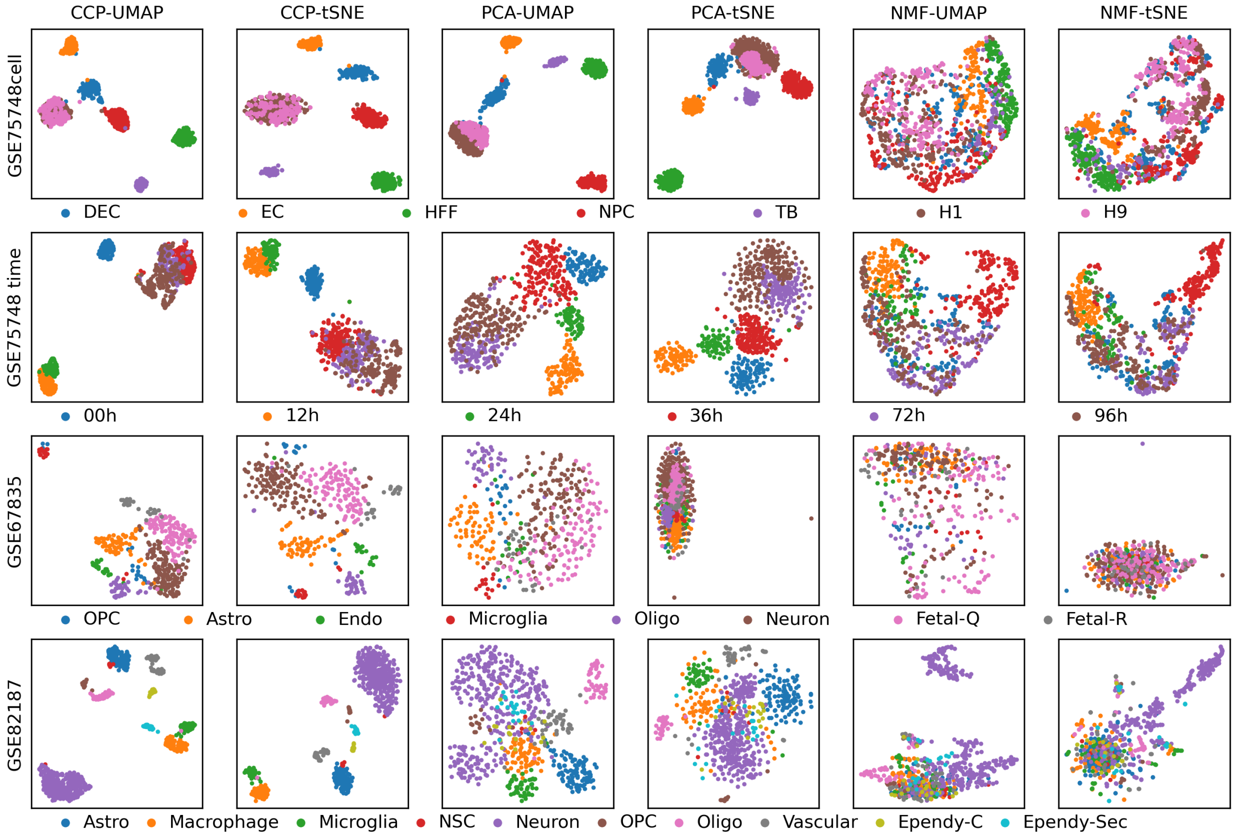

Figure S1: Comparison of CCP-assisted visualization with PCA-assisted and NMF-assisted visualization on GSE75748 cell, GSE75748 time, GSE67835 and GSE82187 data. The columns from left to right show CCP-assisted UMAP, CCP-assisted tSNE, PCA-assisted UMAP, PCA-assisted tSNE, NMF-assisted UMAP and NMF-assisted tSNE. The rows from top to bottom show GSE75748 cell, GSE75748 time, GSE67835 and GSE82187 data. CCP, PCA and NMF were utilized to reduce the dimension to 300, and UMAP and tSNE were used to further reduce the dimension to 2. Sample were colored according to the cell types provided by the original authors.

Figure S2 show the comparison of CCP-assisted, PCA-assisted and NMF-assisted visualization on Quake data. The columns from left to right correspond to CCP-assisted UMAP, CCP-assisted tSNE, PCA-assisted UMAP, PCA-assisted tSNE, NMF-assisted UMAP and NMF-assisted tSNE. Qx indicates scRNA-seq obtained used 10x genomic platform, and Qs indicate data obtained from SmartSeq2 platform.

NMF-assisted visualization do not provide a meaningful clustering result for all data. We can observe the following from the visualization:

- CCP-assisted and PCA-assisted show similar clustering result for both Qx Bladder and Qx Limb data. The most notable difference is that CCP-assisted UMAP show 3 subclusters of bladder urothelial cells.
- In Qs Limb Muscle, both CCP-assisted and PCA-assisted show a similar result.
- In Qs Trachea data, CCP-assisted visualizations show better clustering result from PCA-assisted counterparts. Most notably, the mesenchmyal cells show a stronger clustering in the CCP-assisted visualizations.

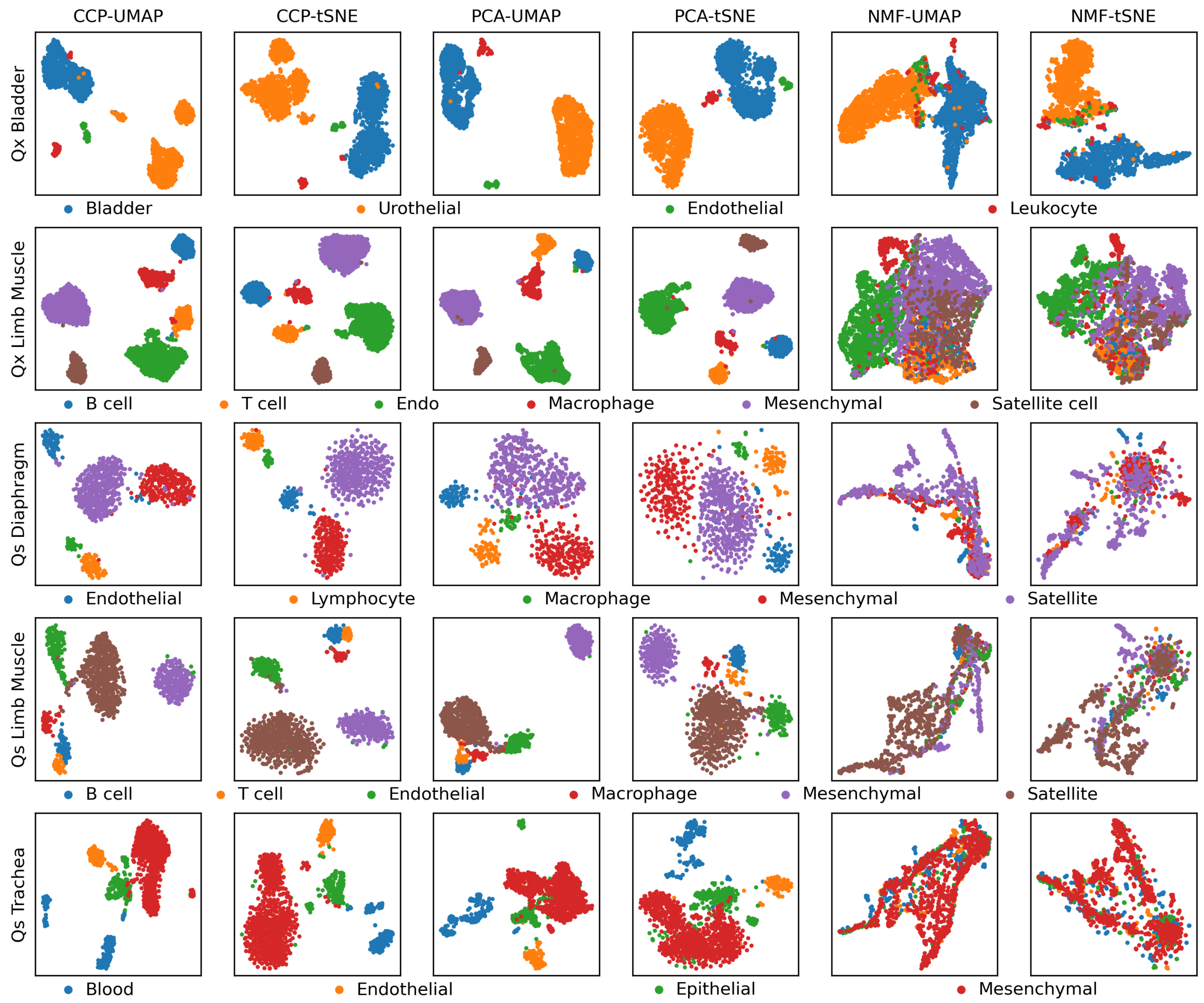

Figure S2: Comparison of CCP-assisted visualization with PCA-assisted and NMF-assisted visualization on Quake data. The columns from left to right show CCP-assisted UMAP, CCP-assisted tSNE, PCA-assisted UMAP, PCA-assisted tSNE, NMF-assisted UMAP and NMF-assisted tSNE. The rows correspond to 1 of the 5 Quake data. Qx indicates scRNA-seq obtained used 10x genomic platform, and Qs indicate data obtained from SmartSeq2 platform. CCP, PCA and NMF were utilized to reduce the dimension to 300, and UMAP and tSNE were used to further reduce the dimension to 2. Sample were colored according to the cell types provided by the original authors.

Figure S3 show the comparison of CCP-assisted, PCA-assisted and NMF-assisted visualization on GSE84133 human data. The columns from left to right correspond to CCP-assisted UMAP, CCP-assisted tSNE, PCA-assisted UMAP, PCA-assisted tSNE, NMF-assisted UMAP and NMF-assisted tSNE. NMF-assisted visualization do not provide a meaningful clustering result for all data. In general, CCP-assisted visualization show the clearest distinction between the cell types. PCA-assisted UMAP and tSNE do not show the distinction between all the cell types.

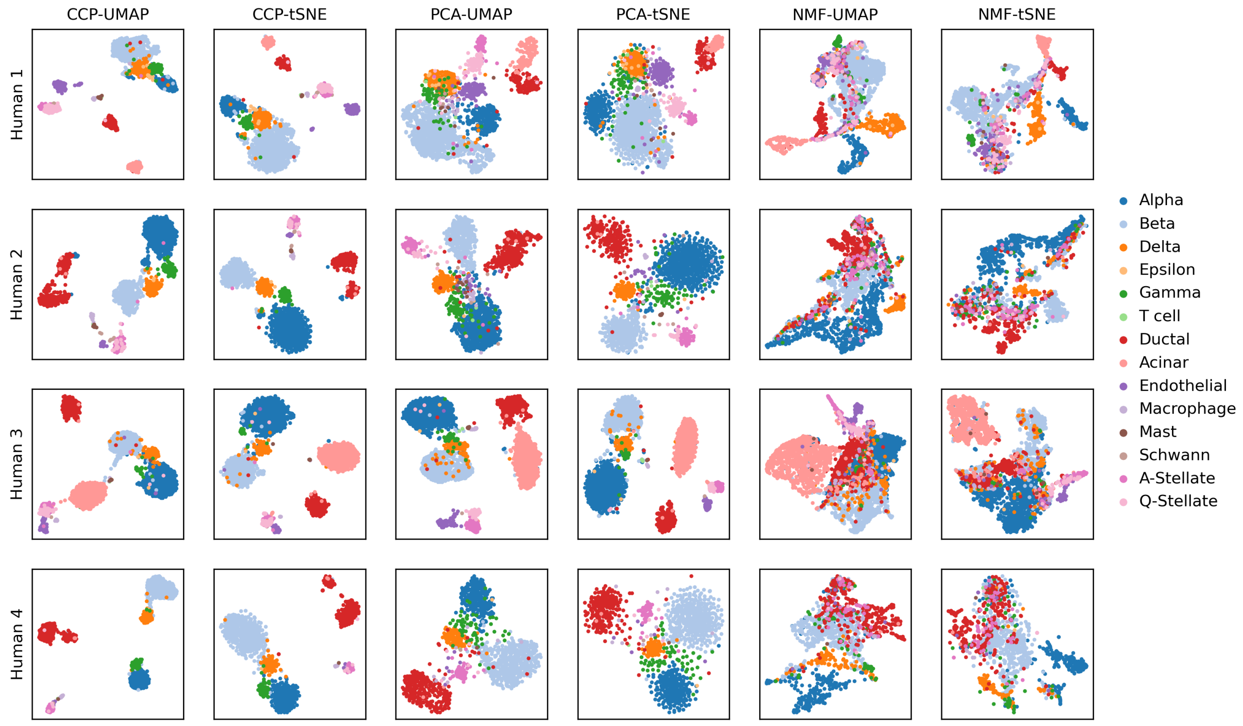

Figure S3: Comparison of CCP-assisted visualization with PCA-assisted and NMF-assisted visualization onGSE84133 human data. The columns from left to right show CCP-assisted UMAP, CCP-assisted tSNE, PCA-assisted UMAP, PCA-assisted tSNE, NMF-assisted UMAP and NMF-assisted tSNE. The rows correspond to one of the four patients. CCP, PCA and NMF were utilized to reduce the dimension to 300, and UMAP and tSNE were used to further reduce the dimension to 2. Sample were colored according to the cell types provided by the original authors.

Figure S4 show the comparison of CCP-assisted, PCA-assisted and NMF-assisted visualization on GSE84133 mouse data. The columns from left to right correspond to CCP-assisted UMAP, CCP-assisted tSNE, PCA-assisted UMAP, PCA-assisted tSNE, NMF-assisted UMAP and NMF-assisted tSNE.

NMF-assisted visualization do not provide a meaningful clustering result for all data. In general, CCP-assisted visualization show the clearest distinction between the cell types. PCA-assisted UMAP and tSNE do not show the distinction between all the cell types.

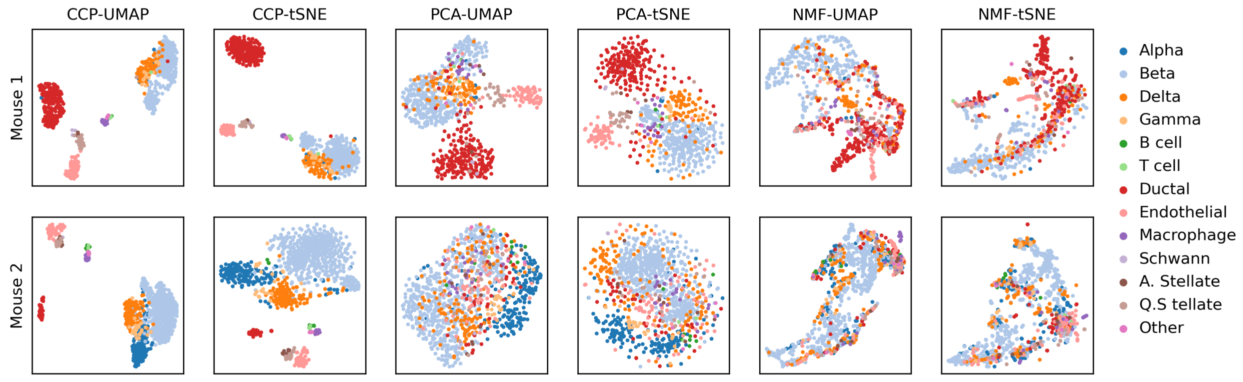

Figure S4: Comparison of CCP-assisted visualization with PCA-assisted and NMF-assisted visualization onGSE84133 human data. The columns from left to right show CCP-assisted UMAP, CCP-assisted tSNE, PCA-assisted UMAP, PCA-assisted tSNE, NMF-assisted UMAP and NMF-assisted tSNE. The rows correspond to mouse 1 and 2. CCP, PCA and NMF were utilized to reduce the dimension to 300, and UMAP and tSNE were used to further reduce the dimension to 2. Sample were colored according to the cell types provided by the original authors.

Figure S5 show the comparison of CCP-assisted, PCA-assisted and NMF-assisted visualization on Muraro, Romanov and Qs Lung data. The columns from left to right correspond to CCP-assisted UMAP, CCP-assisted tSNE, PCA-assisted UMAP, PCA-assisted tSNE, NMF-assisted UMAP and NMF-assisted tSNE.

NMF-assisted visualization do not provide a meaningful clustering result for all data. CCP-assisted and PCA-assisted visualizations are comparable in all the data. In PCA-assisted visualization of Qs Lung, it shows a clear distinction between monocytes and cillated columnar cells, whereas CCP-assisted visualization show a supercluster of these 2 cells. However, the stromal cells in CCP-assisted visualization show a distinct cluster, whereas PCA-assisted show a subcluster within the endothelial cell cluster.

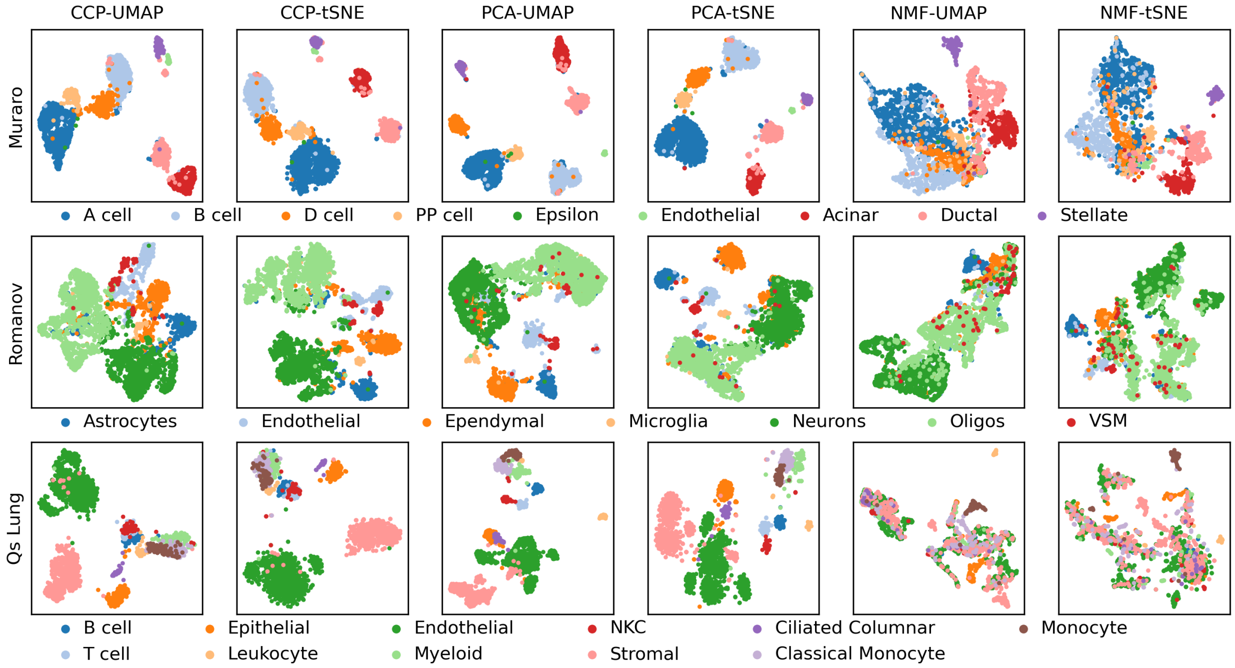

Figure S5: Comparison of CCP-assisted visualization with PCA-assisted and NMF-assisted visualization on Muraro, Romanov and Qs Lung data. The columns from left to right show CCP-assisted UMAP, CCP-assisted tSNE, PCA-assisted UMAP, PCA-assisted tSNE, NMF-assisted UMAP and NMF-assisted tSNE. The rows from top to bottom show Muraro, Romanov and Qs Lung data. CCP, PCA and NMF were utilized to reduce the dimension to 300, and UMAP and tSNE were used to further reduce the dimension to 2. Sample were colored according to the cell types provided by the original authors.

S2.3 Accuracy

In order to validate CCP's performance, we computed the ARI, NMI and ECM for the 18 dataset. For each data, 10 random seed were utilized to generate CCP, PCA and NMF features, and the reduced features were further reduced to 2D using UMAP and tSNE. Leiden clustering was performed to obtain the clustering results, and ARI, NMI and ECM were computed by comparing the clustering results with the cell types provided by the original authors.

Figure S6 show the average ARI, NMI and ECM of CCP-assisted, PCA-assisted and NMF-assisted UMAP and tSNE over 18 dataset. Notice that CCP outperforms both PCA and NMF. CCP-assisted UMAP improves PCA-assisted UMAP on average by 36% in ARI, 21% in NMI and 19% in ECM, and CCP-assisted tSNE improves PCA-assisted tSNE on average by 19% in ARI, 24% in NMI and 18 % in ECM.

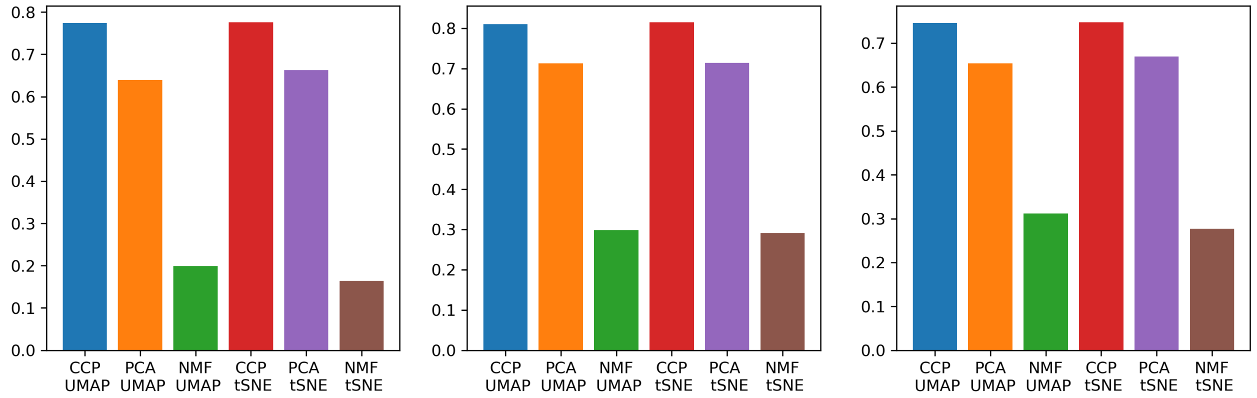

(a) Average ARI over 18 dataset (b) Average NMI over 18 dataset (c) Average ECM over 18 dataset

Figure S6: The average ARI, NMI, ECM of 18 datasets. 10 random initialization was used to compute the reduction for each data. Leiden clustering was used to obtain the clustering results.

Additionally, we validated the CCP super-genes with PCA-gene and NMF-gene. Figure S7 show the average ARI, NMI and ECM of CCP, PCA and NMI over 18 dataset. Notice that CCP outperforms both PCA and NMF. Most notably, CCP improves PCA by 30% in ARI, 20% in NMI and 16% in ECM.

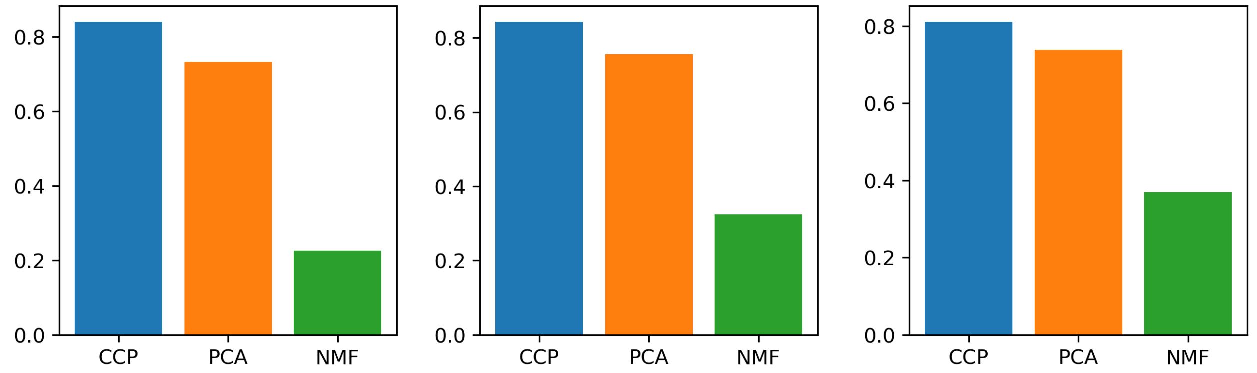

(a) Average ARI over 18 dataset (b) Average NMI over 18 dataset (c) Average ECM over 18 dataset

Figure S7: The average ARI, NMI, ECM of 18 datasets. 10 random initialization was used to compute the reduction for each data. Leiden clustering was used to obtain the clustering results.

CM.

Additionally, we compared CCP-assisted, PCA-assisted and NMF-assisted UMAP and tSNE clustering accuracy for each dataset. Table S2, Table S3 and Table S4 show the ARI, NMI and ECM of each data, respectively. CCP-assisted UMAP and tSNE outperforms the the other methods on almost all the data we have tested.

Table S2: ARI of the clustering result for each data

Data	CCP UMAP	CCP tSNE	PCA UMAP	PCA tSNE	NMF UMAP	NMF tSNE	UMAP	tSNE
GSE75748cell	0.768	0.772	0.761	0.776	0.273	0.268	0.814	0.890
GSE75748time	0.561	0.557	0.636	0.723	0.222	0.229	0.656	0.706
GSE82187	0.947	0.953	0.430	0.575	0.157	0.114	0.720	0.660
GSE67835	0.739	0.791	0.365	0.251	0.068	0.020	0.630	0.658
GSE84133human1	0.765	0.833	0.639	0.765	0.257	0.213	0.497	0.668
GSE84133human2	0.877	0.879	0.819	0.828	0.268	0.223	0.894	0.909
GSE84133human3	0.920	0.922	0.917	0.927	0.414	0.353	0.826	0.847
GSE84133human4	0.862	0.866	0.817	0.893	0.151	0.134	0.942	0.945
GSE84133mouse1	0.707	0.726	0.537	0.562	0.269	0.215	0.451	0.513
GSE84133mouse2	0.709	0.772	0.194	0.227	0.118	0.066	0.282	0.329
Muraro	0.823	0.753	0.702	0.746	0.222	0.210	0.567	0.789
Romanov	0.515	0.532	0.548	0.543	0.253	0.209	0.559	0.630
Quake_10x_Bladder	0.623	0.602	0.538	0.513	0.459	0.304	0.653	0.785
Quake_10x_Limb_Muscle	0.947	0.848	0.897	0.888	0.393	0.340	0.905	0.973
Quake_Smart-seq2_Diaphragm	0.947	0.957	0.839	0.827	0.059	0.043	0.788	0.929
Quake_Smart-seq2_Limb_Muscle	0.916	0.921	0.885	0.913	0.106	0.090	0.859	0.937
Quake_Smart-seq2_Lung	0.811	0.830	0.591	0.606	0.078	0.072	0.762	0.784
Quake_Smart-seq2_Trachea	0.618	0.606	0.518	0.515	0.046	0.037	0.479	0.503

Table S3: NMI of the clustering result for each data

Data	CCP UMAP	CCP tSNE	PCA UMAP	PCA tSNE	NMF UMAP	NMF tSNE	UMAP	tSNE
GSE75748cell	0.901	0.907	0.895	0.895	0.402	0.409	0.926	0.928
GSE75748time	0.704	0.697	0.773	0.830	0.325	0.325	0.788	0.848
GSE82187	0.937	0.946	0.616	0.648	0.284	0.290	0.773	0.676
GSE67835	0.789	0.811	0.507	0.346	0.177	0.083	0.710	0.785
GSE84133human1	0.807	0.826	0.710	0.753	0.412	0.413	0.624	0.695
GSE84133human2	0.859	0.872	0.777	0.783	0.339	0.357	0.853	0.872
GSE84133human3	0.883	0.886	0.890	0.896	0.436	0.436	0.790	0.808
GSE84133human4	0.864	0.864	0.771	0.829	0.264	0.274	0.906	0.909
GSE84133mouse1	0.769	0.791	0.589	0.608	0.316	0.321	0.574	0.604
GSE84133mouse2	0.747	0.769	0.266	0.288	0.196	0.192	0.403	0.460
Muraro	0.820	0.802	0.815	0.831	0.352	0.362	0.641	0.785
Romanov	0.592	0.613	0.591	0.603	0.284	0.295	0.560	0.591
Quake_10x_Bladder	0.740	0.727	0.711	0.690	0.505	0.442	0.742	0.811
Quake_10x_Limb_Muscle	0.949	0.903	0.930	0.915	0.489	0.468	0.927	0.948
Quake_Smart-seq2_Diaphragm	0.906	0.925	0.827	0.791	0.187	0.176	0.808	0.907
Quake_Smart-seq2_Limb_Muscle	0.858	0.867	0.837	0.854	0.235	0.234	0.793	0.891
Quake_Smart-seq2_Lung	0.794	0.805	0.751	0.746	0.211	0.216	0.798	0.805
Quake_Smart-seq2_Trachea	0.748	0.740	0.660	0.633	0.114	0.122	0.590	0.609

Table S4: ECM of the clustering result for each data

Data	CCP UMAP	CCP tSNE	PCA UMAP	PCA tSNE	NMF UMAP	NMF tSNE	UMAP	tSNE
GSE75748cell	0.901	0.907	0.793	0.794	0.332	0.329	0.831	0.849
GSE75748time	0.704	0.697	0.677	0.766	0.310	0.319	0.692	0.736
GSE82187	0.937	0.946	0.504	0.610	0.254	0.246	0.652	0.608
GSE67835	0.789	0.811	0.435	0.333	0.220	0.208	0.600	0.631
GSE84133human1	0.807	0.826	0.586	0.686	0.352	0.316	0.485	0.580
GSE84133human2	0.859	0.872	0.778	0.787	0.334	0.299	0.850	0.860
GSE84133human3	0.883	0.886	0.871	0.886	0.451	0.367	0.786	0.811
GSE84133human4	0.864	0.864	0.775	0.843	0.274	0.261	0.894	0.896
GSE84133mouse1	0.769	0.791	0.591	0.605	0.374	0.340	0.518	0.545
GSE84133mouse2	0.747	0.769	0.353	0.398	0.253	0.204	0.391	0.415
Muraro	0.820	0.802	0.712	0.756	0.328	0.316	0.578	0.767
Romanov	0.592	0.613	0.532	0.513	0.306	0.252	0.556	0.622
Quake_10x_Bladder	0.740	0.727	0.541	0.516	0.487	0.317	0.647	0.779
Quake_10x_Limb_Muscle	0.949	0.903	0.893	0.878	0.431	0.354	0.890	0.963
Quake_Smart-seq2_Diaphragm	0.906	0.925	0.829	0.812	0.233	0.243	0.786	0.924
Quake_Smart-seq2_Limb_Muscle	0.858	0.867	0.851	0.876	0.267	0.250	0.803	0.910
Quake_Smart-seq2_Lung	0.794	0.805	0.558	0.567	0.214	0.188	0.659	0.673
Quake_Smart-seq2_Trachea	0.748	0.740	0.560	0.543	0.215	0.213	0.505	0.529

S3 Discussion

S3.1 Large Data

Figure S8 show additional example of utilizing subsampling to handle large data. The details of the procedure can be found in Section 4.1 of the main text. Figure S8(a) shows the ARI and NMI under increasing subsampling amount. Additionally, Figure S8(b) shows the CCP-assisted UMAP and tSNE under varying subsampling amount. Similar to GSE84133 human and Qx Spleen data, CCP is stable under the subsampling procedure, and the consistency of the CCP-assisted visualizations under varying subsampling procedure indicate robustness of the method. For the computation time, subsampling scheme using 1000 samples took 171 seconds.

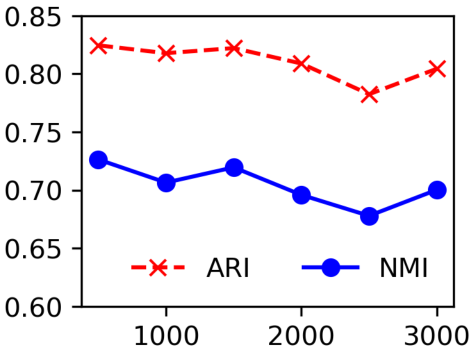

(a) ARI and NMI under different subsampling.

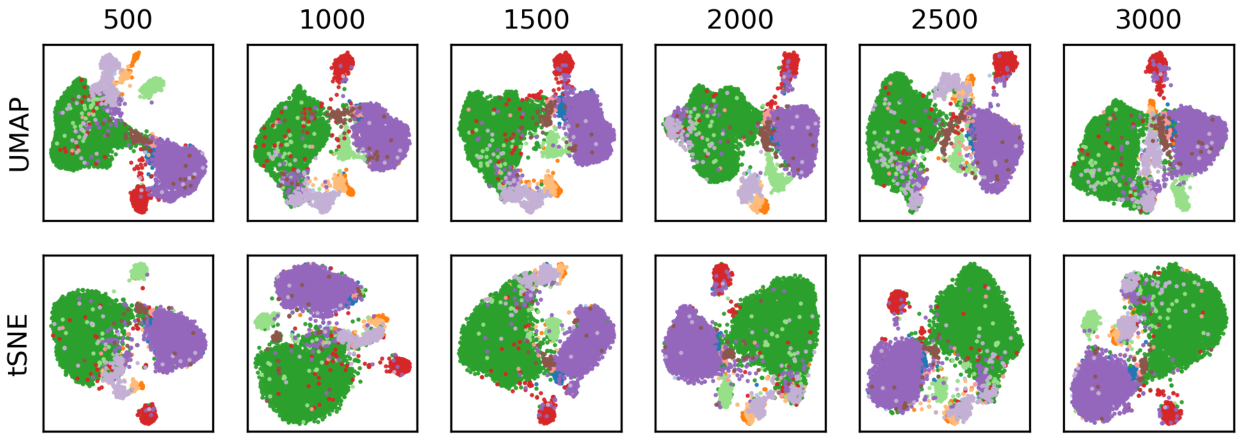

(b) CCP-assisted UMAP and tSNE visualization of SCP1749

Figure S8: UMAP and tSNE visualization of SCP17249 data under different number of subsampling. 300 super-genes were generated from CCP, and Leiden clustering was used to obtain the clustering results. (a) ARI and NMI under different subsampling values of SCP1749 data. (b) CCP-assisted UMAP and tSNE of SCP1749 data under different number of subsampling.

S3.2 Low variance gene

Figure S9 show the effect of varying the number of super-genes and the cutoff ratio on the predictive power and visualization of GSE75748cell data. We utilized 10 random seeds to generate CCP super-genes using different number of super-genes and cutoff ratio. Then, Leiden clustering was used to obtain the cluster labels, and the ARI was computed by using the cell types provided by the original authors. Notice that at all cutoff ratio, the ARI increases as the number of super-genes increases, and the ARI is comparable at 300 super-genes. This suggest the robustness of LV-gene. Additionally, we computed the number of genes in the LV-gene cluster at varying cutoff value, and plotted with the variance of the genes in descending order. Notice that at $\nu_c = 0.9$ the variance of the genes are relatively small, indicating that the predictive power of these genes may not be high.

Figure S9(c) show the visualization of CCP-assisted UMAP and tSNE at various cutoff ratio. For the visualization, 300 super-genes were utilized, and UMAP and tSNE was applied to the super-genes to reduce the dimension to 2. Samples were then colored according to the cell types provided by the original authors. Note that all the visualization are comparable, indicating the robustness of LV-gene under different cutoff ratio.

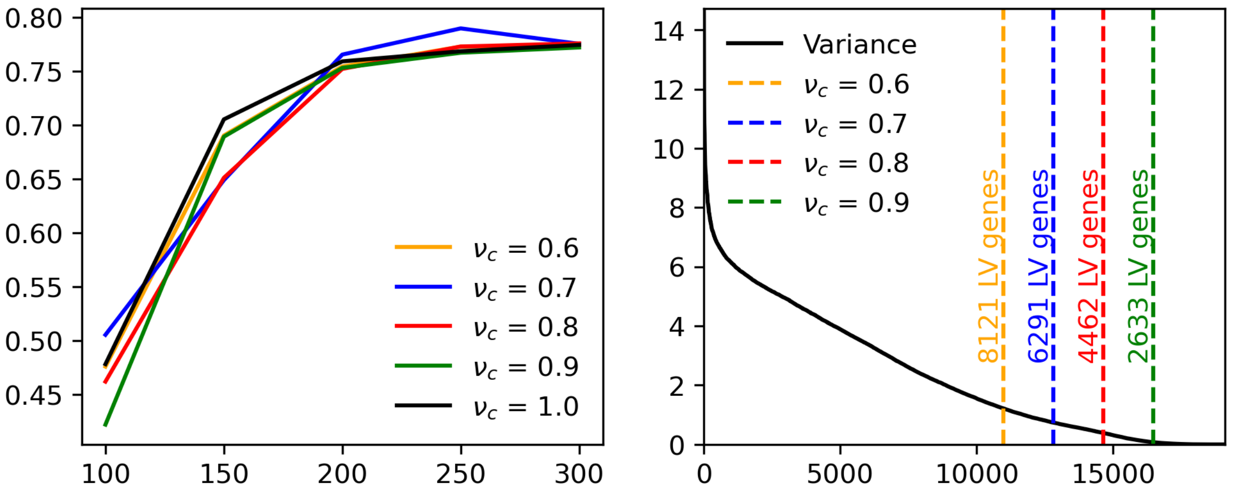

(a) ARI of number of super-genes and ν_c

(b) Number of genes in LV genes

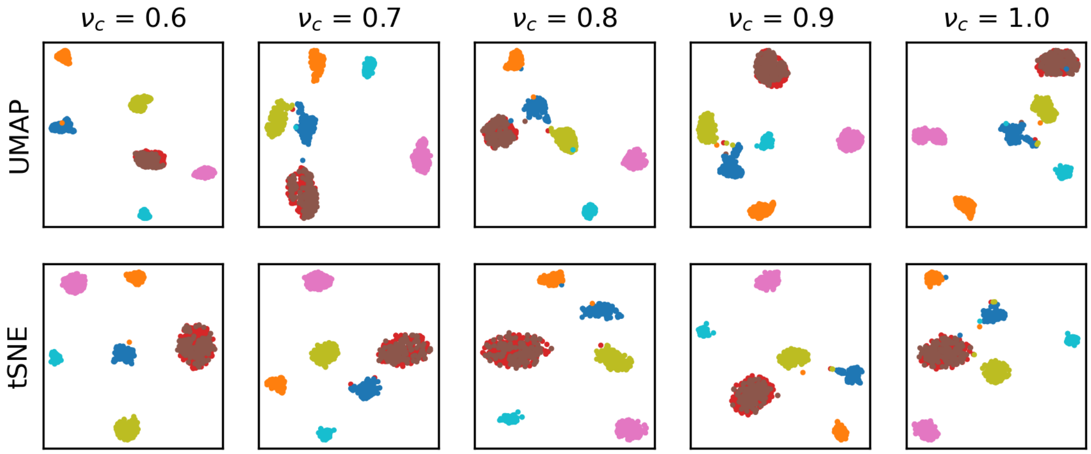

(c) CCP-assisted UMAP and tSNE visualization

Figure S9: Analysis of varying the cutoff ratio ν_c on clustering and visualization of GSE75748 cell data. (a) ARI of leiden clustering when the number of super-genes and cutoff ratio is changed. (b) The number of genes in the LV-gene when ν_c is changed. (c) Top and bottom row shows the CCP-assisted UMAP and t-SNE visualization, and the columns corresponds to $\nu_c = 0.6, 0.7, 0.8, 0.9$. 300 super-genes were used to initialize UMAP and tSNE, and the samples were colored according to the true cell type.

References

[1] Li-Fang Chu, Ning Leng, Jue Zhang, Zhonggang Hou, Daniel Mamott, David T Vereide, Jeea Choi, Christina Kendziorski, Ron Stewart, and James A Thomson. Single-cell rna-seq reveals novel regulators of human embryonic stem cell differentiation to definitive endoderm. *Genome biology*, 17:1–20, 2016.
