# Supplementary material for: Analyzing scRNA-seq data by CCP-assisted UMAP and tSNE
Source: PLoS One. 2024 Dec 13;19(12):e0311791. doi: 10.1371/journal.pone.0311791 (PMC11642954; doi:10.1371/journal.pone.0311791)
Supplement: Attachment — Submitted filename: Response to Reviewers.pdf [file pone.0311791.s002.pdf]

Comment From Editor

Comment 1: *Please ensure that your manuscript meets PLOS ONE's style requirements, including those for file naming.*

Answer 1: We have followed the submission formatting guidelines

Comment 2: *Please note that PLOS ONE has specific guidelines on code sharing for submissions in which author-generated code underpins the findings in the manuscript. In these cases, all author-generated code must be made available without restrictions upon publication of the work.*

Answer 2: All the code used to generate our results is submitted to Github https://github.com/hozumiyu/CCP-scRNAseq-UMAP-TSNE

Comment 3: *Thank you for stating the following financial disclosure. Please state what role the funders took in the study.*

Answer 3: The funders had no role in study design, data collection and analysis, decision to publish, or preparation of the manuscript. We have indicated this in the cover letter.

Comment 4: *Please expand the acronym "NIH, NSF, NASA and MSU" (as indicated in your financial disclosure) so that it states the name of your funders in full. This information should be included in your cover letter; we will change the online submission form on your behalf.*

Answer 4: NIH: National Institute of Health, NSF: National Science Foundation, NASA: National Aeronautics and Space Administration, MSU: Michigan State University

Comment 5: *Thank you for stating the following in the Acknowledgments Section of your manuscript: "This work was supported in part by NIH grants R01GM126189, R01AI164266, and R35GM148196 , NSF grants DMS-2052983, DMS-1761320, and IIS-1900473, NASA grant 80NSSC21M0023, MSU Foundation, Bristol-Myers Squibb 65109, and Pfizer." We note that you have provided funding information that is not currently declared in your Funding Statement. However, funding information should not appear in the Acknowledgments section or other areas of your manuscript. We will only publish funding information present in the Funding Statement section of the online submission form. Please include your amended statements within your cover letter; we will change the online submission form on your behalf.*

Answer 5: We have removed the acknowledgement section in our manuscript. The current funding statement is appropriate, and no further addition is neccessary.

Comment 6: *Please remove your figures from within your manuscript file, leaving only the individual TIFF/EPS image files, uploaded separately. These will be automatically included in the reviewers' PDF.*

Answer 6: We have removed the figures from the manuscript.

Reviewer 1

Comments: *Authors utilize the proposed method CCP as an initialization tool for uniform manifold approximation and projection (UMAP) and t-distributed stochastic neighbor embedding (tSNE) and reported enhancement of CCP assisted UMAP and tSNE over UMAP and tSNE. The paper is well written, organised and I believe the propose method may be good alternative for scRNA-seq data visulaization. I have some suggestions and comments givn below:*

Answer We appreciate the reviewer for the work.

Comment 1: *Nowadays, many deep learning-based dimensionality reduction methods are introduced and they show promising results. It is not clear what are the advantages of the proposed methods over those deep learning-based methods apart from CCP has the ability to work on small size datsets. Some comparison results of CCP assisted UMAP and tSNE, along with PCA and NMF and deep learning assisted UMAP and tSNE may be reported.*

Answer 1: Although deep learning models have become prevalent in scRNA-seq analysis, they may give very little insight to what are happened within the deep learning models. CCP, similar to NMF, performs the reduction purely in the data domain, and also preserves the nonnegativity of the original data. Such method is much more interpretable than deep-learning models. Additionally, our visualization approach is targeted for single-experiment reduction, whereas deep learning models often need careful curation of multiple data sources. Our approach has been compared with UMPA and tSNE, showing the improvement.

Comment 2:*Nowadays, scRNA-seq datasets contain millions of cells. Authors have considered datasets size less than 10000 cells. I suggest to incorporate results on some large datasets.*

Answer 2: The goal of the subsampling procedure was to tackle larger data. We have added additional data SCP1749 from the Broad Institute's single cell repository, which consists of more than 10,000 cells. The result was added to the supporting materials. We are developing a GPU version of CCP which enables us to deal with millions of cells (however, this is out of the scope of the present paper).

Comment 3:*Authors have reported some impressive results. However, apart from average NMI and ARI reported in Fig 7, detailed results on each of the methods in terms of ARI, NMI on each datasets is expected in tabular format may be in supplementary.*

Answer 3: We have added the results for each data as a table in the supporting materials section S1.3.1.

Comment 4: *In scRNA-seq data analysis running time is also another issue. It would be helpful for reader if authors can report a comparison of the proposed method in terms of running time and memory usage. I also suggest to incorporate systems specification on which the method was tested.*

Answer 4: We benchmarked our method using Michigan State University's high performance computing cluster, which utilized AMD EPYC 7H12 Processor. For all the test, we used 1 CPU core with 16gb of memory. We have added the description into the manuscript. As mentioned, with a GPU version, the benchmark will change.

Comment 5: *The result reported in Fig 8 can be extended by considering a datset of much larger size.*

Answer 5: We have added additional data SCP1749 from the Broad Institute's single cell repository, which consists of more than 10,000 cells. The result was added to the supporting materials.

Comment 6: *I wanted access to the method online. But the link is not working.*

Answer 6: The code to replicate our results can be found at https://github.com/hozumiyu/CCP-scRNAseq-UMAP-TSNE

Comment 7: *Typos: line number 236*

Answer 7: We have fixed the typo on line 236 14$ to 14%

Comment 8: *Need captions in supporting figures in supplementary.*

Answer: We have added captions to all the figures in the supporting materials

Reviewer 2

Comments: *The authors proposed to utilize correlated clustering and projection to analyze scRNA-seq data. The work may have been a laborious one that I should appreciate the authors' time and patient to come up with some results. However, there are several problems that deduct from the quality of this manuscript. Below are several comments on this work.*

Comment 1: *The article does not reflect the time consumption performance of this method. Is it suitable for large-scale data sets (Data of more than 10,000 cells)? Are there any disadvantages compared to standard UMAP or t-SNE?*

Answer 1: Because the method requires distance computation for each super-genes, the time-complexity is proportional to the number of super-genes and the distance calculation. For larger-data, using a subset of the data to train CCP will reduce the computational time, and an analysis is performed in the discussion. Standard UMAP and tSNE require distance calculation, which is difficult for high dimensional data (i.e., curse of dimensionality). CCP overcomes this limitation by reducing the original dimension to 300 super-gene. Moreover, the distance calculation is much more reliable using the CCP, resulting in a much better result. We are developing GPU and multiple CPU based CCP methods, which will enable us to deal with millions of cells.

Comment 2: *Is it scientific that the Urothelial in the Qx Bladder data set in Figure 2 is divided into three sub-clusters? Is it biologically significant?*

Answer 2: This is an interesting question. The real cause may be multiple. For example, the data may have contaminated. There may be batch effect. One needs to carefully investigate the original data collection protocol before assuming a biological significance, which is beyond the scope of our work.

Comment 3: *Is it scientific for Mesenchymal in the Qx Trachea data set in Figure 2 to cluster two sub-clusters into one category?*

Answer 3: Currently, no method is able to correctly classify all single cells, due to its limitation or noisy experimental data. This is reason that the research community is continuously developing new methods.

Comment 4: *Ductal in the Human4 data set in Figure 4 is divided into two clusters in CCP-assisted. Is this biologically meaningful?*

Answer 4: As stated early, the real cause may be multiple. For example, the data may have contaminated. There may be batch effect. One needs to carefully investigate the original data collection protocol before assuming a biological significance, which is beyond the scope of our work.

Reviewer 3

Comments 1: *English expressions need to be edited more careful and more native, in this manuscript, there are some mistakes. For example, "The The samples were colored according to the true cell type" in line 205 on page 13.*

Answer 1: We have gone through additional rounds of editing to fix these mistakes.

Comments 2: *I suggest the authors should add a flowchart in the manuscript to show the process very well.*

Answer 3: We have added a flowchart.

Comments 3: *Important computational models about dimensionality reduction and feature selection in scRNA-seq analysis should be cited. Some recommended studies are helpful (PMIDs: 36642414, 36924730, and 37660567).*

Answer: We appreciate additional reference, and have incorporated them into the introduction.

Comments 4: *The authors should carefully check and unify the information of references. Some references lack the information of volume or contain the wrong page number.*

Answer: We have gone through each reference to ensure that the references are correct and made the reference more consistent.

Reviewer 4

Comments: *Correlated Clustering and Projection (CCP) is a data dimensionality reduction method previously designed by the authors. During the dimensionality reduction process, CCP separately processed the low variance (LV) genes. In this manuscript, the authors found that the reduced data obtained from CCP dimensionality reduction can improve the visualization effect of UMAP and tSNE. From the authors' experiments, the effect is good.*

Comment We thank the reviewer for the positive feedback.

Comments 1: *CCP first clusters genes, which may mask the importance of different genes. Some clusters may be sets of low expression genes, while some others may be sets of high expression genes. Some marker genes are originally unique, but they may be in the same cluster as other non-marker genes, or there may be many marker genes in a certain cluster, or some clusters may not have any marker genes. That is, during the gene clustering process, the uniqueness of marker genes may be reduced? Please reply if there are any possibilities that I mentioned here.*

Answer 1: It is certainly possible for the marker genes to be clustered with non-marker genes as long as these genes exhibit similar pattern as the marker genes. We would like to emphasize that CCP is entirely unsupervised. We have not incorporated prior knowledge for the super-gene, but we would like to consider guided feature partition using prior knowledge in the future.

Comments 2: *It is best to draw a flowchart or an example diagram for the method. Solely using descriptions and formulas can confuse us on subtle differences, such as whether x_m^n in line 105 and x_i^n in formula (5) represent the same thing.*

Answer 2: We have added a flowchart to better clarify our methodology.

Comments 3: *The authors set the number of super-genes to 300 for all datasets, but the number of genes in the original datasets used in the paper is different. Why not dynamically adjust the number of super-genes based on the number of genes?*

Answer 3: We fixed the number of super-genes to be 300 to be entirely parameter-free in our analysis. Additionally, for potential use in the deep learning data preprocessing, a fixed number of output is needed. However, it would be up to the user to adjust the number of super-gene as well as the LV-gene for the particular dataset.

Comments 4: *P5, line 118, What weights are these w_{im} and how to set?*

Answer 4: We have set $w_{im} = 1$ in this work. We would like to further explore this parameter to incorporate other information, such as spatial information in spatial transcriptomics.

Comments 5:*P4 line 104, Does $z_m^{S^n}$ represent the expression vector of the corresponding cluster?*

Answer 5: $z_m^{S^n}$ represents the super-gene n for the m-th cell.

Comments 6: *Page10 Line 167, dataa –¿data*

Answer 6: We have fixed the typo.

Reviewer 5

Comments 1: *Provide a more detailed explanation of how CCP partitions genes and identifies super-genes to enhance understanding.*

Answer 1: We have added more details to clarify the methodology. Additionally, we added a flowchart to clarify the method.

Comments 2: *Include specific details about CCP implementation and parameter settings for reproducibility.*

Answer 2: We have used the same parameters for all the tests. We set $\kappa = 2$, $\tau = 6$ and $n = 300$. We have added the choice of parameter in the manuscript. We have also added these explanation in the github repository.

Comments 3: *Compare CCP with other dimensionality reduction techniques such as PCA or autoencoders to contextualize its performance.*

Answer 3: The comparison with PCA and NMF has been carried out and can be found in the supporting materials.

Comments 4: *Detail the statistical tests used to claim improvements in UMAP and tSNE, including p-values, to substantiate your results.*

Answer 4: We have added additional table in the supporting materials that show the ARI, NMI and ECM of each data we have tested.

Comments 5: *Describe the 18 datasets used, including their sources and characteristics, to assess the generalizability of the findings.*

Answer 5 In the supporting materials, we have a table characterizing the dataset, which include the host species, and statistics on the expression profiles.

Comments 6: *Discuss how CCP specifically handles the sparsity in scRNA-seq data, a critical challenge in the field.*

Answer 6: Sparsity is indeed a challenge that every method faces when working with scRNA-seq data. In our method, we utilize LV-gene to collect low-varying genes to obtain a stronger signal. Additionally, CCP partitions genes according to their similarity (i.e., similar sparsity patterns in sparse genes).

Comments 7: *Explain the biological relevance of the improvements made by CCP to highlight its impact on the field.*

Answer 7: Gene expression has many correlations. For example, genes belonging to the same pathway may exhibit similar expression patterns. CCP identifies these patterns in an unsupervised fashion (i.e., does not need prior knowledge). Since no prior knowledge is utilized, our method provides additional perspective in scRNA-seq analysis in an unbiased fashion.

Comments 8: *Provide information about any software available for implementing CCP to aid in adoption and further research.*

Answer 8: Code and data availability is described Sec. 6.

Comments 9: *Identify limitations of CCP and suggest areas for future research to guide subsequent studies.*

Answer 9: The limitation of CCP is in the computational time. We have proposed subsampling to circumvent this limitation, and discussed the stability under subsampling in the Discussion section.

In the future, we would like to extend the work to spatial data. In particular, the weights w_{im} can incorporate prior information, such as spatial information of the cell. Additionally, the current CCP is entirely unsupervised. We would like to incorporate marker genes as a prior to guide in the gene-partitioning procedure in the future. We have added these comments in the manuscript. Finally, we are working on GPU and parallel versions of CCP.

Comments 10: *Improve the quality of graphical representations of UMAP and tSNE results to better communicate your findings.*

Answer 10 We have made the dots smaller in our figures to see the clusters more clearly.

Comments 11: *Incorporate recent literature to position CCP within the current research landscape and ensure the study is up-to-date. Advancing single-cell RNA-seq data analysis through the fusion of multi-layer perceptron and graph neural network. iDNA-OpenPrompt: OpenPrompt learning model for identifying DNA methylation*

Answer 11: We appreciate the additional reference. We have added the suggested reference into the introduction.

Reviewer 6

Comment *In this manuscript, the authors propose the usage of the Correlated Clustering and Projection (CCP) method as an initiation step for t-SNE and UMAP. They have presented results which show substantial improvement over ordinary t-SNE and UMAP when CCP is used as a preprocessing step. However, there are a few concerns.*

Major concerns: ——————

Comments 1: *The CCP algorithm used in this work cannot be considered as a benchmark since it is not yet published as a peer-reviewed journal or as a part of any conference proceedings.*

Answer 1: CCP has been published, see doi: 10.1021/acs.jcim.3c00674.

Comments 2: *The authors have not mentioned what values of parameters they have used for t-SNE and UMAP. As it is quite well known that these parameters (for e.g. perplexity in t-SNE or n_neighbors and min-dist in UMAP) are very sensitive, probably fine-tuning them could have achieved the same result, which is not clear. Infact, the codebase https://github.com/hozumiyu/CCP-scRNAseq-UMAP-TSNE/blob/main/main.py reveals the usage of one pair of values for the dataset GSE57249 and another pair of values for others. No particular guideline for setting these values have been provided.*

Answer 2: We have updated the github repository with a much more detailed tutorial. We have utilized the default parameters of UMAP and tSNE, as outlined in the original UMAP package, as well as the sklearn implementation of tSNE. These values are n_neighbors = 15 and perplexity = 45.

Comments 3: *Again, based on the tutorial notebook provided in github, it is not clear why a z-score normalization is required before running UMAP, even when the dataset is preprocessed using CCP. The authors have not presented any discussion about it in the manuscript.*

Answer 3: We apologize for the confusion. Our most up-to-date method does not utilize any normalization after CCP. We have updated the github repository with a much more thorough tutorial.

Comments 4: *The comparative results shown in this manuscript are mainly qualitative. For quantitative comparison, the authors have used only three measures, namely ARI, NMI and ECM. However, they have reported an average over 18 datasets (Figures 7 and S7). This is not an acceptable result at all. Should we assume that results for the 18 datasets have a large variation? Also, how to access performance in situations where ground truth labels are not available? In addition to ARI, NMI, ECM, pairwise distance preservation before and after CCP-UMAP or CCP-tSNE could be measured.*

Answer 4: We have added an additional table to show the ARI, NMI and ECM for each data.

Comments 5:*In section 4, the authors mention "Large" datasets. However, the largest dataset used in this study does not have more than 10k cells. Considering technological advancements in the field of scRNA-seq, a million cells is now a reality. So, questions remain as to what is a really large amount? The authors should give an estimate of the running time requirement for the subsample-based strategy for such a dataset.*

Answer 5: We have added another data that has over 10,000 cells in the supporting materials. Additionally, we included the computational time. On average, it took 152 seconds, 160 seconds and 171 seconds for GSE84133 human, Qx Spleen and SCP1749 data, respectively. We are developing GPU and parallel versions of CCP, which will further speed up the method.

Minor: ———

Comments 6: *ECM - Please change "Element-centeric" to "Element-Centric"*

Answer 6: We have fixed the manuscript

Comments 7: *Page 16 - $ instead of %*

Answer 7: We have fixed the manuscript

Comments 8: *"Section ??" - Section reference missing in S1.2 in supporting material*

Answer 8: We have fixed the manuscript

Comments 9: *Page 8 - S1.3 - $ instead of %*

Answer 9: We have fixed the manuscript
